# Knowledge, Attitude, and Practice of Vital Pulp Therapy Among Dental Professionals in Taif, Saudi Arabia: A Cross-sectional Study

**DOI:** 10.4317/jced.63083

**Published:** 2025-11-30

**Authors:** Hassan T. Shawli, Yazeed M. Althomali, Bandar S. Shukr, Hatim A. Alqarni, Ibrahim D. Althobaiti, Mohammed S. Alosaimi

**Affiliations:** 1 Department of Restorative and Dental Sciences, Faculty of Dentistry, Taif University, P.O. Box 11099, Taif 21944, Saudi Arabia; 2 Department of Preventive Dentistry, Faculty of Dentistry, Taif University, P.O. Box 11099, Taif 21944, Saudi Arabia; 3 Faculty of Dentistry, Taif University, P.O. Box 11099, Taif 21944, Saudi Arabia

## Abstract

**Background:**

Vital pulp therapy (VPT) is a key regenerative approach in the field of endodontics and a recognized therapeutic method for permanent teeth by several practice organizations. This study aimed to examine the knowledge and attitudes toward VPT in permanent teeth, and the adoption of the updated practice guidelines among dentists in Taif, Saudi Arabia.

**Material and Methods:**

Data were collected from 197 general dentists and specialists practicing in Taif using an anonymous questionnaire. It comprised four sections: demographics, questions regarding the indications and diagnosis of VPT (Domain 1), VPT for immature permanent teeth (Domain 2), and the different materials and restorations utilized in VPT (Domain 3). The data were analyzed using descriptive statistics and linear regression.

**Results:**

80% of the participants possessed adequate knowledge in Domain 1; however, misconceptions were evident (83.2%) regarding the limitations of VPT in patients with reversible pulpitis. The knowledge levels were lower in Domains 2 and 3 (73%) compared with Domain 1. Misconceptions were common (69.5%) regarding the appropriate timing for permanent coronal restoration placement and the diagnostic role of sodium hypochlorite. Furthermore, age and workplace significantly influenced VPT knowledge, with higher knowledge observed among young dentists (aged 25-34 years) and lower knowledge noted among private sector dentists.

**Conclusions:**

Although most dentists exhibited acceptable knowledge and attitudes, gaps were evident in case selection, restoration timing, and material application. Targeted education and ongoing training are needed, particularly for private sector dentists and those with limited experience, to improve endodontic care in the Taif region.

## Introduction

The dental pulp tissue in the coronal and radicular areas is the living component of the tooth, and its vitality is essential for the tooth's long-term survival (1). The pulp tissue is highly sensitive to various stimuli that can jeopardize its vitality (1). Examples of these stimuli include microbial invasion through dental caries, mechanical or chemical irritations during dental procedures, and dental trauma (1). These stimuli can trigger the pulpal inflammatory process, which may progress to pulpal necrosis and loss of vitality (1). Vital pulp therapy (VPT) is one of the key regenerative areas in the field of endodontics (2). This biological therapeutic approach was developed to maintain the health and integrity of the pulp tissue that has been compro¬mised but not destroyed by different stimuli (3 , 4). VPT procedures involve indirect or direct pulp capping as well as partial or complete pulpotomy (5). VPT preserves pulp vitality in immature permanent teeth, facilitating continued root development. This method is indicated for traumatic pulp exposures when histological analysis shows that pulp inflammation is limited to the superficial layers (6). The condition of the pulp and the level of root development are critical factors that determine the success of VPT (7). For mature permanent teeth, VPT is recommended for mechanical or traumatic pulp exposures only if the diagnosis is "reversible pulpitis" without any signs of periapical pathologies (8). This clinical practice was adopted for several years and has changed only recently. Histological studies have observed that in cases of carious pulp exposure, the infection is often confined to the coronal chamber of the root canal system and that the radicular pulp tissue does not exhibit any signs of inflammation (9 , 10). Therefore, if the carious lesion is removed and the infected pulp tissue is amputated, the remaining healthy tissue has the potential to be repaired (3). Recently, Wolters et al. proposed a new classification for the diagnosis of pulpitis (11). The authors stated that areas of uninflamed pulp tissue may be present even in patients diagnosed with "irreversible pulpitis." Hence, they recommended the use of VPT to treat these cases. In addition, a recent review suggested the use of VPT as a potential treatment alternative in mature permanent teeth with carious pulp exposure and a pulpal diagnosis of "irreversible pulpitis" (3). This recommendation agrees with the recently updated guidelines published by the American Association of Endodontists (AAE) (12) and the European Society of Endodontology (ESE) (13). Dental caries is highly prevalent in Saudi Arabia. A recent systematic review reported a prevalence of approximately 84% among children aged 5-7 years and 72% among those aged 12-15 years (14). This high caries pattern can lead to the early loss of permanent first molars at a young age, exerting adverse effects on both skeletal and dental development (15). Moreover, dental trauma is a prominent concern among children in Saudi Arabia, with a documented prevalence of 39.5%-44% in studies conducted in different Saudi regions (16 - 18). Furthermore, Al-Madi et al. identified that 36.9% of Saudi children and adolescents aged 6-18 years had immature permanent posterior teeth with pulpal involvement (19). Hence, the treating dentists should be familiar with diverse VPT techniques and be able to select the most appropriate one to save the involved tooth and ensure complete root development in immature teeth. VPT remains an area of debate regarding its diagnostic criteria, the condition of the pulp at the time of treatment, the most effective therapeutic approach, and the overall prognosis (20). Nonetheless, to ensure successful treatment outcomes, VPT practice guidelines have been proposed by several well-established organizations, including the AAE, the American Association of Pediatric Dentistry, and the ESE. These guidelines are continuously updated based on insights from ongoing research. Currently, limited evidence is available regarding VPT knowledge and the implementation of updated practice guidelines among dental practitioners in Saudi Arabia, especially in the Taif region. To date, only one published study has evaluated VPT knowledge among practitioners at an international dental conference in Riyadh (1). However, this study was performed before the release of the most recent practice guidelines (before 2021). Therefore, this research aimed to assess the knowledge and attitudes toward VPT in permanent teeth as well as the adoption of updated clinical practice guidelines among dental practitioners in Taif, Saudi Arabia.

## Material and Methods

- Study Design and Ethical Considerations This survey-based, descriptive, cross-sectional study was conducted in a convenience sample of licensed general dental practitioners (GDPs) and specialists in Taif, Saudi Arabia, using a self-administered, structured online questionnaire. The study was conducted between October 2024 and December 2024. A consent statement was included in the first part of the questionnaire, and participants were considered to have provided their consent if they agreed to participate and completed the questionnaire. Ethical approval was obtained from the Scientific Research Ethics Committee of Taif University, Taif, Saudi Arabia (Approval No. 46-059). Additionally, this manuscript was prepared in accordance with the 2023 guidelines of the Preferred Reporting Items for Observational Studies in Endodontics (PROBE) (21). - Study Sample This study focused on all licensed dentists practicing in Taif, Saudi Arabia. As per the 2023 statistics published by the Ministry of Health (MOH) in Saudi Arabia, the Taif Governorate has 758 licensed dentists, including 117 in MOH hospitals, 94 in MOH primary health care centers, and 547 in private sector facilities (i.e., clinics, hospitals, and medical complexes) (22). The "Raosoft" website was used to calculate the sample size after considering a 5% margin of error and a 95% confidence level, and the estimated sample was found to be a minimum of 256 participants. Dentists (both specialized and nonspecialized) who were licensed, currently practicing in Taif, and willing to participate in the study were included. Dental students, dental interns, and other dental personnel were not eligible to participate. To account for nonresponse bias, 280 dentists were invited to participate and complete the study survey. However, 78 of the invited dentists refused to participate. In addition, five respondents were excluded from the study as they were not practicing in Taif during the time of data collection. Consequently, the final analytical sample comprised 197 dental practitioners. - Data Collection Instrument (Study Questionnaire) Data were collected using an anonymous, self-administered, structured survey developed using Google Forms. The survey comprised 23 validated, multiple-choice questions adapted from the study by Doumani et al. (1). Furthermore, certain modifications were applied after consulting two experienced endodontists affiliated with the same academic institution, in addition to reviewing recent VPT publications (3 , 23 - 25) and incorporating the updated practice guidelines (12 , 13). All questions were in English and designed in a closed-ended format. Although previously validated, the internal consistency of the modified questionnaire was reassessed using Cronbach's alpha, yielding a score of 0.82. To minimize bias, the survey was pilot-tested for validation on six dental practitioners before data collection. The feedback suggested that some of the survey questions needed further clarification; hence, these items were refined, and the responses from the pilot-testing sample were not included in the final study sample. The survey was structured into four sections, each designed to collect different types of information. Each study participant took approximately 8-10 min to complete the survey. To minimize information bias expected in self-administered survey studies, the questionnaire began with an overview of the research and its objectives, highlighting the significance of providing accurate responses. Moreover, a consent statement was included to ensure data confidentiality and inform participants of their right to withdraw at any time. The first section of the survey included questions related to demographics and clinical practice. The subsequent three sections comprised 16 items that evaluated the knowledge and practice of VPT among the study participants in the following three domains: Domain 1 (three items related to the indications and diagnosis of VPT); Domain 2 (six items on different vital pulp therapies for immature permanent teeth); and Domain 3 (seven items concerning different materials and restorations used in VPT). A one-point score was given for each correct answer in each domain, and the total VPT knowledge scores were calculated for each domain as well as for all three domains combined. The highest achievable knowledge score was 16, while the lowest possible score was zero if a respondent answered all the questions incorrectly. - Statistical Analysis All data were exported into a Microsoft Excel spreadsheet and analyzed using the Statistical Package for the Social Sciences software (Chicago, IL, USA, version 25.0). Descriptive statistics, including frequency distribution and percentages, were computed for all survey items, as appropriate. Furthermore, the effects of distinct demographic parameters on VPT knowledge in the three domains and the overall VPT knowledge were assessed using adjusted multiple linear regression. The results from regression analysis were expressed as 95% confidence intervals (CIs) and adjusted beta coefficients. Residual plots were constructed to confirm the homogeneity of variances, whereas the Kolmogorov-Smirnov test and histogram plots were utilized to evaluate data normality. The variance inflation factor was used to detect potential multicollinearity among the study variables. Additionally, Cook's distance was employed to identify any significant outliers. To determine the models' goodness of fit, the adjusted coefficient of determination (Adj. R²) was applied. No violations were found regarding these assumptions in any of the models. Moreover, collapsing was performed on groups containing a small number of participants, as necessary. All statistical tests were conducted using a two-tailed approach, and a p-value of 0.05 was considered statistically significant.

## Results

A total of 202 dental practitioners participated in the study. Most (97.5%) were currently practicing in Taif, with Saudi nationals constituting 76.2% of the participants. The age distribution indicated that 58.9% were aged 25-34 years, followed by 27.2% aged 24 years, 12.4% aged 35-44 years, and 1.5% aged 45 years. GDPs represented the largest group (62.4%), whereas 9.1% were endodontic specialists and 28.4% were nonendodontic specialists. Regarding work status, 38.6% were employed in the governmental sector, 21.8% in the private sector, and 39.6% were residents. A high proportion of the participants (57.9%) had 1-5 years of professional experience, and only 0.5% had &gt;20 years of experience (Table 1).

Table 1

**Figure 1:**
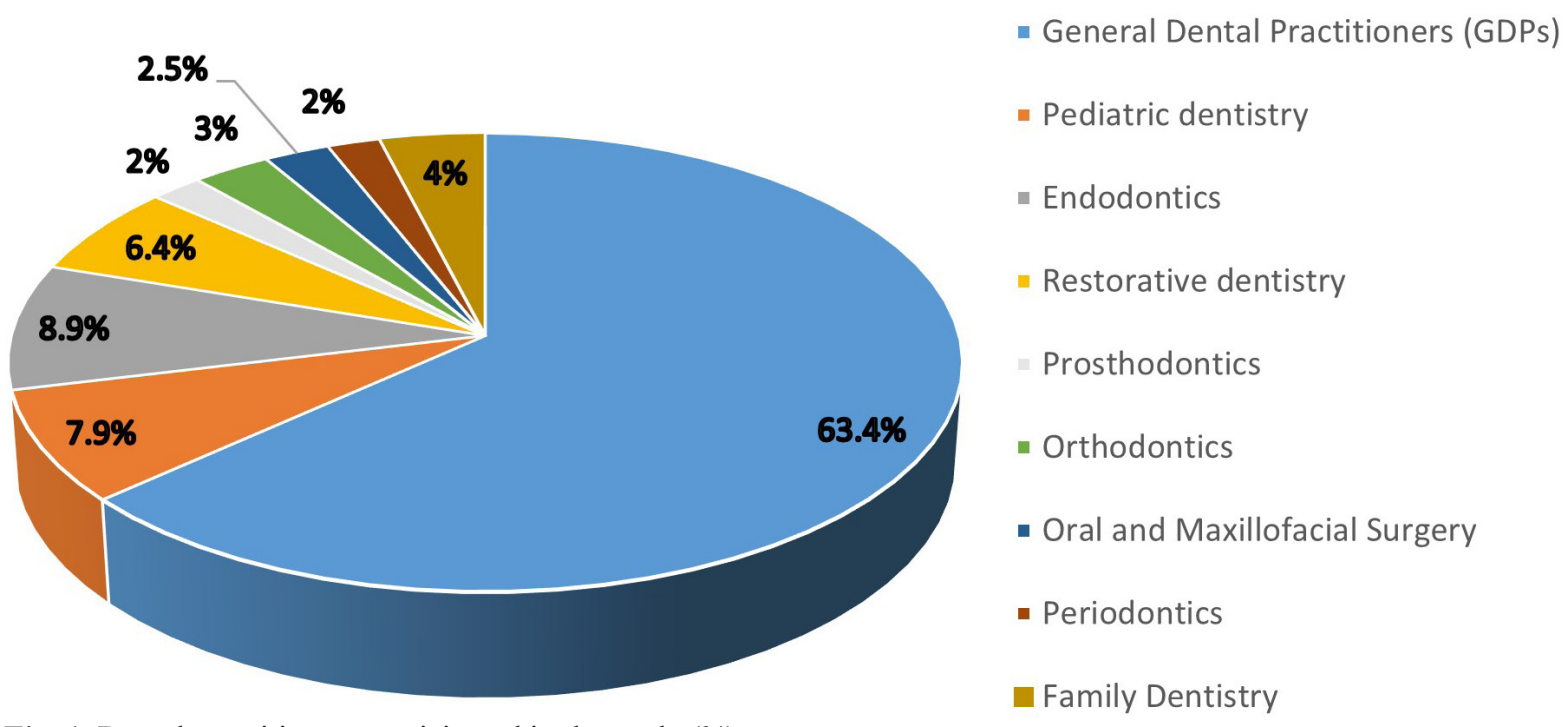
Dental practitioners participated in the study (%).

The distribution of dental professionals across different specializations is presented in Figure 1.

1

GDPs constituted the most significant proportion, accounting for 63.4% of the total, emphasizing their role in dental care. Endodontists represented 8.9% of the dental workforce, followed by pediatric dentists at 7.9%. Restorative dentists and family dentists comprised 6.4% and 4% of the total, respectively. Orthodontists and oral and maxillofacial surgeons constituted 3% and 2.5% of the total sample, respectively. Periodontists and prosthodontists each represented 2% of the total dental professionals. A total of 197 participants were included in the analytical sample. In terms of knowledge and practices related to VPT, most participants (89.3%) correctly identified that electric and thermal pulp tests might be unreliable after traumatic injuries. Nonetheless, 83.2% wrongly agreed that VPT should only be performed in teeth with reversible pulpitis, and 80.2% correctly identified the primary objective of VPT as initiating the formation of tertiary reparative dentin or a calcific bridge (Domain 1). Regarding VPT procedures in immature permanent teeth (Domain 2), 83.2% correctly recognized apexogenesis as a method to encourage root end development and 84.4% agreed that it maintains pulp vitality for continued dentin deposition. Nevertheless, only 74.6% correctly identified that the process facilitates dentine bridge formation at the pulpotomy site. Moreover, 83.8% correctly identified the role of apexification in forming a calcified barrier in roots with an open apex. Awareness of indirect pulp capping procedures was high, with 77.7% correctly identifying their purpose. However, most respondents (69.5%) had incorrect knowledge regarding the recommended follow-up period for permanent restoration placement. In Domain 3, concerning dental materials used in VPT and restorations, 81.7% correctly acknowledged the drawbacks of calcium hydroxide and 75.6% recognized the superior properties of mineral trioxide aggregate (MTA) for pulpal repair. In addition, 76.6% agreed that if bleeding cannot be controlled within 10 min after partial pulpotomy, complete coronal pulp removal is preferred. Nonetheless, the diagnostic role of sodium hypochlorite was incorrectly understood by 69.5% of the respondents. Approximately 74% agreed that VPT success rates decrease with increasing patient age, and 77.2% supported the use of caries detector dyes in conservative caries removal. Lastly, 80.2% correctly identified that substituting MTA with calcium hydroxide does not significantly alter apical maturation timelines (Table 2).

Table 2

**Figure 2:**
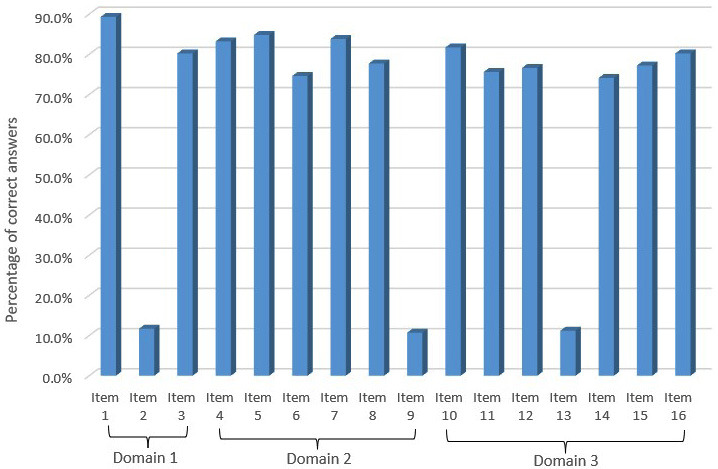
VPT Knowledge categories across the different questionnaire items in each domain (%).

Figure 2 presents the percentages of correct answers across all items. Overall, the accuracy rates varied across items, with most items achieving a high percentage of correct responses and a few items demonstrating a low accuracy.

2

In Domain 1, Item 1 exhibited the highest percentage of correct responses (&gt;90%), followed by Item 3. However, Item 2 showed a significantly lower percentage, with an accuracy rate of &lt;20%, indicating potential difficulty or misinterpretation. Domain 2 followed a similar trend, with most items demonstrating accuracy rates of &gt;70%. Items 4, 5, and 7 displayed high accuracy levels, approaching 85%-90%. However, Item 9 exhibited a considerable drop in performance, with an accuracy of &lt;20%, implying increased difficulty or conceptual misunderstanding among respondents. Domain 3 showed relatively consistent performance across items, with the accuracy of most items being &gt;70%. However, Item 13 demonstrated a noticeable decline, with an accuracy of &lt;20%, mirroring the pattern observed in Domains 1 and 2. Overall, the findings suggest that while most items were answered correctly by a large proportion of respondents, specific items in each domain exhibited significantly lower accuracy, warranting further investigation into potential sources of difficulty. Table 3 lists the associations between demographic parameters and knowledge of VPT in each of the three domains and all three domains combined.

Table 3

Nationality did not demonstrate any significant associations; nonetheless, a borderline effect was noted in the overall model, with higher VPT knowledge among non-Saudi dentists ( = 0.88, 95% CI [0.17, 1.93], p = 0.099). Dentists aged 25-34 years possessed significantly higher knowledge in Domain 2 ( = 0.55, 95% CI [0.09, 1.01], p = 0.021), Domain 3 ( = 0.75, 95% CI [0.16, 1.34], p = 0.013), and the overall score across all domains ( = 1.46, 95% CI [0.45, 2.47], p = 0.005) compared with younger dentists (24 years). In contrast, participants aged 35 years did not show significant differences in VPT knowledge relative to the youngest age group in any of the domains. Regarding specialty, endodontic and nonendodontic specialists did not demonstrate significantly different knowledge levels compared with GDPs in any of the domains. Dentists working in the private sector obtained significantly lower knowledge scores in Domain 1 ( = 0.31, 95% CI [0.56, 0.06], p = 0.015), Domain 3 ( = 0.93, 95% CI [1.58, 0.27], p = 0.006), and the overall score across all domains ( = 1.62, 95% CI [2.74, 0.51], p = 0.005) compared with those employed in the governmental sector. Participants with &gt;10 years of experience showed higher VPT knowledge in all domains and the overall score compared with less experienced dentists (1-5 years), but these associations were not statistically significant.

## Discussion

The primary goal of VPT is to preserve pulpal health in teeth affected by trauma, caries, restorative treatments, or anatomical irregularities by encouraging pulpal cells to generate dentin, creating a long-lasting seal that maintains the integrity of the pulp (26 - 30). Furthermore, VPT plays a crucial role in protecting immature permanent teeth with incomplete root formation (31). The findings of this study provide valuable insights into the knowledge, attitudes, and practices of dental professionals regarding VPT. Additionally, the study is significant as it is the first to investigate this topic in the population of licensed dentists in Taif, Saudi Arabia. The observations revealed that although most participants were adequately knowledgeable in VPT principles, significant gaps remain in certain areas, necessitating further education and training. According to the study findings, 58.9% of the participants were aged 25-34 years, 62.4% were GDPs, and 57.9% had 1-5 years of experience. These figures imply that the participants were predominantly early-career general practitioners, which may influence both clinical preferences and treatment patterns. The participants' overall knowledge regarding the indications and diagnosis of VPT (Domain 1) was higher (80% and above) than that reported in a study by Doumani et al. (1) targeting dental practitioners in Riyadh, Saudi Arabia. Nonetheless, misconceptions were evident, particularly regarding limiting the use of VPT to cases of reversible pulpitis, emphasizing the need for enhanced understanding. This finding may reflect outdated training based on earlier practice guidelines that recommended the use of VPT only in the case of reversible pulpitis. The guidelines on case selection for VPT were recently updated, (12 , 13) recommending its use in both reversible and irreversible pulpitis. The use of VPT in immature permanent teeth to induce physiological processes (i.e., apexogenesis and apexification) was also assessed in this investigation (Domain 2). Apexogenesis is a histological term describing the ongoing natural development and formation of the root apex (32). This technique is used to stimulate the formation of a calcified barrier in a root with an open apex or to promote further apical development in an incompletely formed root of a tooth with necrotic pulp (33). The dental professionals included in this research exhibited relatively higher (73%) knowledge levels in this domain compared with those enrolled in the study by Doumani et al. (1). In addition, approximately 77% of the participants agreed that indirect pulp capping can be applied in teeth with deep carious lesions approximating the pulp but without signs or symptoms of pulp degeneration. This percentage is higher than that reported in the study by Rabi, which surveyed dental practitioners in the country of Palestine (59.7%) (34). However, a misconception was observed regarding the appropriate timing for placing a permanent coronal restoration in the case of indirect pulp capping. This observation can also be explained by the lack of awareness of most practitioners about the recent update in the practice guidelines. Similar to Domain 2, the dental practitioners in this study demonstrated higher (73%) knowledge regarding the dental materials and restorations used in VPT (Domain 3) than those who participated in the research by Doumani et al. (1). Nonetheless, the misinterpretation of sodium hypochlorite's diagnostic role suggests a gap in understanding the material's broad applications. In terms of the clinical application of VPT, this study supports previous reports indicating that MTA is widely regarded as the most effective material for pulpal repair (35). However, the lack of consensus on the disadvantages of calcium hydroxide suggests that outdated beliefs persist, emphasizing the significance of ongoing professional education. Studies have established that although MTA offers better biocompatibility and sealing properties, calcium hydroxide remains in use despite its drawbacks, including poor long-term sealing and material degradation (36 , 37). The association of demographic characteristics with VPT knowledge provided further insights. Younger dentists (aged 25-34 years) obtained significantly higher knowledge scores than their older counterparts, suggesting that recent graduates might have been exposed to more up-to-date curricula and training. The considerably lower knowledge levels among private sector dentists highlight the effect of workplace environment and access to continuous education. This finding could be ascribed to the enhanced access to educational resources available for governmental sector practitioners, such as updated clinical guidelines and training programs. Moreover, the lack of significant knowledge differences between endodontic specialists and other dental practitioners implies that fundamental VPT concepts are broadly understood across specialties; however, these may not be entirely integrated into specialized practice. These findings underscore the importance of targeted educational initiatives in bridging specific knowledge gaps. Practitioners' confidence in VPT case selection and material application can be strengthened by incorporating updated VPT guidelines into dental curricula and continuous education programs. In addition, reinforcing evidence-based clinical protocols via workshops and training sessions can augment the practical implementation of VPT. One suggestion is to incorporate modular Continuing Medical Education (CME) programs (38), and case-based seminar formats (39 , 40) that are focused on different endodontic topics. These formats allow for greater flexibility and practical application, catering to diverse learning preferences among dental professionals. Modular CME can offer asynchronous learning opportunities, while case-based seminars encourage critical thinking and peer discussion around real-world scenarios. Both approaches can enhance clinical decision-making and promote adherence to best practices in endodontic care. Despite the significance of this study, certain limitations exist. First, the study could not achieve the required minimum sample size owing to the high number of invited dentists who declined to participate. Second, the gender effect could not be evaluated as most respondents who agreed to participate and answered the study survey were men (97%). Hence, women participants were excluded from the study sample to avoid the issue of participation or nonresponse bias. Third, the cross-sectional design limits the ability to confirm causal relationships. Additionally, self-reported responses may introduce some bias. Fourth, the findings could not be generalized to all dental practitioners in Saudi Arabia because of the convenience sampling technique and the fact that only practitioners in Taif were enrolled. Finally, although the study highlighted significant effects for certain factors (such as age and work status), some were found to have a borderline effect (such as nationality, specialty, and years of experience). All these findings warrant further studies, with larger samples and a sufficient number of participants in relevant categories, to better understand VPT and related factors.

## Conclusions

Notwithstanding the overall high levels of knowledge regarding VPT among the sampled dental practitioners, deficiencies were noted in critical areas, including case selection, the appropriate timing for placing permanent restorations, and material application. The findings emphasize the need for targeted educational programs and continuous professional training to enhance the quality of endodontic care in Taif and to bridge the existing knowledge gaps, particularly among professionals in the private sector and those with limited clinical experience.

## Figures and Tables

**Table 1:** Table Characteristics of the study participants (N = 202).

Characteristic	Category	n	%
Currently practicing in Taif	Yes	197	97.5
	No	5	2.5
Nationality	Saudi	154	76.2
	Non-Saudi	48	23.8
Age (years)	24 years or less	55	27.2
	25–34 years	119	58.9
	35–44 years	25	12.4
	45 years or older	3	1.5
Specialty	General Dental Practitioners (GDPs)	128	62.4
	Endodontic specialists	18	9.1
	Non-endodontic specialists	56	28.4
Work status	Governmental sector	78	38.6
	Private sector	44	21.8
	Resident	80	39.6
Experience (years)	1–5 years	117	57.9
	5–10 years	62	30.7
	10–15 years	17	8.4
	15–20 years	5	2.5
	More than 20 years	1	0.5

1

**Table 2:** Table Participants’ response to different VPT domains (N = 197).

Item	n	%
After traumatic injuries, electric and thermal pulp tests may be unreliable		
Yes	176	89.3
No	11	5.6
I don’t know	10	5.1
VPT should only be performed in teeth with reversible pulpitis		
Yes	164	83.2
No	23	11.7
I don’t know	10	5.1
The main objective in VPT is to initiate the formation of tertiary reparative dentin or calcific bridge		
Yes	158	80.2
No	21	10.7
I don’t know	18	9.1
Apexogenesis is a VPT procedure to encourage the physiological development and formation of the root end		
Yes	164	83.2
No	22	11.2
I don’t know	11	5.6
Apexogenesis maintains pulp vitality thus allows continued deposition of dentin		
Yes	167	84.4
No	16	8.1
I don’t know	14	7.1
Apexogenesis allows generating dentine bridge at the site of pulpotomy		
Yes	147	74.6
No	22	11.2
I don’t know	28	14.2
Apexification is a method to induce a calcified barrier in a root with open apex		
Yes	165	83.8
No	12	6.1
I don’t know	20	10.2
Indirect pulp capping is a procedure performed in a tooth with a deep carious lesion approximating the pulp but without signs or symptoms of pulp degeneration		
Yes	153	77.7
No	25	12.7
I don’t know	19	9.6
In indirect pulp capping, the patient returns in 8-12 weeks for placement of a permanent coronal restoration		
Yes	137	69.5
No	21	10.7
I don’t know	39	19.8
The drawbacks of Calcium hydroxide [Ca (OH)2] include weak marginal adaptation to dentin, and dissolution over time		
Yes	161	81.7
No	19	9.6
I don’t know	17	8.6
The unique physiochemical properties of Mineral Trioxide Aggregate (MTA) promote a superior environment for pulpal repair and bridge formation, compared to Calcium hydroxide [Ca (OH)2] products		
Yes	149	75.6
No	21	10.7
I don’t know	27	13.7
In partial or shallow pulpotomy: if bleeding cannot be controlled after 10 minutes of direct exposure to Sodium hypochlorite (NaOCl) after removal of unhealthy tissue, complete removal of the coronal pulp to the pulp floor is the preferred option		
Yes	151	76.6
No	15	7.6
I don’t know	31	15.7
Sodium hypochlorite (NaOCl) serves as an excellent diagnostic tool to differentiate irreversible from reversible pulpitis and to help determine whether to proceed with partial pulpotomy, complete pulpotomy, or pulpectomy		
Yes	137	69.5
No	22	11.2
I don’t know	38	19.3
Successful outcomes for VPT decrease as the patient’s age increase		
Yes	146	74.1
No	21	10.7
I don’t know	30	15.2
Caries detector dyes can be considered a valuable tool in caries excavation when attempts are made to preserve mineralizable dentin and to minimize trauma to the pulp		
Yes	152	77.2
No	20	10.2
I don’t know	25	12.7
If Mineral Trioxide Aggregate (MTA) is substituted for Calcium hydroxide [Ca (OH)2] in VPT procedures, similar time periods for apical maturation can be anticipated		
Yes	158	80.2
No	15	7.6
I don’t know	24	12.2

2

**Table 3:** Associations between demographic parameters and VPT knowledge in different domains (N = 197).

Parameter	Domain 1 (indications and diagnosis) Adj. R² = 0.03		Domain 2 (VPTs for immature permanent teeth) Adj. R² = 0.90		Domain 3 (dental materials and restorations) Adj. R² = 0.24		All domains Adj. R² = 0.22	
	β (95% CI)	P-value	β (95% CI)	P-value	β (95% CI)	P-value	β (95% CI)	P-value
Nationality (Saudi)	Reference	Reference	Reference	Reference	Reference	Reference	Reference	Reference
Nationality (non-Saudi)	0.05 (-0.18 – 0.28)	0.680	0.32 (-0.16 – 0.80)	0.189	0.51 (-0.10 – 1.12)	0.101	0.88 (-0.17 – 1.93)	0.099
Age (24 years or less)	Reference	Reference	Reference	Reference	Reference	Reference	Reference	Reference
Age (25-34 years)	0.16 (-0.06 – 0.39)	0.155	0.55 (0.09 – 1.01)	0.021 *	0.75 (0.16 – 1.34)	0.013 *	1.46 (0.45 – 2.47)	0.005 *
Age (35 years or older)	-0.07 (-0.42 – 0.28)	0.697	0.19 (-0.53 – 0.92)	0.596	-0.66 (-1.58 – 0.26)	0.158	-0.54 (-2.11 – 1.04)	0.503
Speciality (GDPs)	Reference	Reference	Reference	Reference	Reference	Reference	Reference	Reference
Speciality (endodontic specialists)	-0.05 (-0.38 – 0.28)	0.772	0.32 (-0.36 – 1.00)	0.361	0.75 (-0.11 – 1.62)	0.089 ¥	1.02 (-0.47 – 2.51)	0.177
Speciality (non-endodontic specialists)	-0.01 (-0.24 – 0.22)	0.925	0.43 (-0.05 – 0.91)	0.078 ¥	0.55 (-0.06 – 1.16)	0.078 ¥	0.97 (-0.08 – 2.02)	0.069 ¥
Work status (governmental sector)	Reference	Reference	Reference	Reference	Reference	Reference	Reference	Reference
Work status (private sector)	-0.31 (-0.56 – 0.06)	0.015 *	-0.39 (-0.90 – 0.12)	0.136	-0.93 (-1.58 – -0.27)	0.006 *	-1.62 (-2.74 – -0.51)	0.005 *
Work status (resident)	0.20 (-0.19 – 0.60)	0.314	-0.13 (-0.96 – 0.69)	0.752	-0.03 (-1.08 – 1.02)	0.955	0.04 (-1.75 – 1.84)	0.963
Years of experience (1-5 years)	Reference	Reference	Reference	Reference	Reference	Reference	Reference	Reference
Years of experience (5-10 years)	0.09 (-0.14 – 0.33)	0.439	-0.46 (-0.95 – 0.03)	0.064 ¥	-0.48 (-1.01 – 0.15)	0.134	-0.84 (-1.91 – 0.22)	0.120
Years of experience (more than 10 years)	0.28 (-0.08 – 0.63)	0.125	0.01 (-0.71 – 0.74)	0.967	0.26 (-0.67 – 1.19)	0.579	0.55 (-1.04 – 2.14)	0.494

3

## Data Availability

The data used to support the findings of this research are available from the corresponding author upon reasonable request.
